# 2-Chloro-*N*-(4-meth­oxy­benzo­yl)­benzene­sulfonamide

**DOI:** 10.1107/S1600536814001482

**Published:** 2014-01-25

**Authors:** S Sreenivasa, B. S. Palakshamurthy, K. J. Pampa, N. K. Lokanath, P. A. Suchetan

**Affiliations:** a Department of Studies and Research in Chemistry, Tumkur University, Tumkur, Karnataka 572 103, India; b Department of Studies and Research in Physics, U.C.S., Tumkur University, Tumkur, Karnataka 572 103, India; c Department of Studies in Microbiology, University of Mysore, Manasagangotri, Mysore, India; d Department of Studies in Physics, University of Mysore, Manasagangotri, Mysore, India; e Department of Studies and Research in Chemistry, U.C.S., Tumkur University, Tumkur, Karnataka 572 103, India

## Abstract

In the title compound, C_14_H_12_ClNO_4_S, the dihedral angle between the aromatic rings is 82.07 (1)° and the dihedral angle between the planes defined by the S—N—C=O fragment and the sulfonyl benzene ring is 82.46 (3)°. In the crystal, the mol­ecules are linked into *C*(4) chains running along [001] by strong N—H⋯O hydrogen bonds. A C—H⋯O intera­ction reinforces the [001] chains: its graph-set symbol is *C*(7). The chains are cross-linked into (100) sheets by further C—H⋯O inter­actions as *C*(6) chains along [001]. The structure also features weak π–π stacking inter­actions [centroid–centroid distances = 3.577 (1) and 3.8016 (1) Å].

## Related literature   

For related structures see: Gowda *et al.* (2010*a* ▶,*b* ▶); Suchetan *et al.* (2011*a* ▶,*b* ▶).
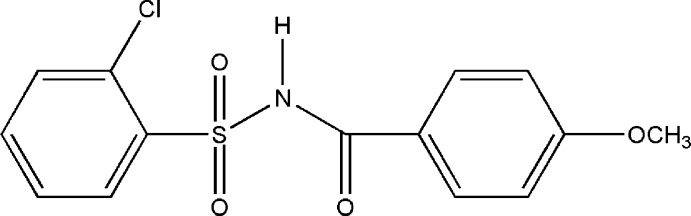


## Experimental   

### 

#### Crystal data  

C_14_H_12_ClNO_4_S
*M* *_r_* = 325.76
Monoclinic, 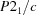

*a* = 14.5293 (19) Å
*b* = 10.6225 (14) Å
*c* = 9.6918 (14) Å
β = 92.695 (5)°
*V* = 1494.2 (4) Å^3^
*Z* = 4
Cu *K*α radiation
μ = 3.71 mm^−1^
*T* = 293 K
0.44 × 0.35 × 0.26 mm

#### Data collection  

Bruker APEXII CCD diffractometer
Absorption correction: multi-scan (*SADABS*; Bruker, 2009 ▶) *T*_min_ = 0.263, *T*_max_ = 0.381
17846 measured reflections
2449 independent reflections
2295 reflections with *I* > 2σ(*I*)
*R*_int_ = 0.058

#### Refinement  

*R*[*F* ^2^ > 2σ(*F* ^2^)] = 0.063
*wR*(*F* ^2^) = 0.163
*S* = 1.11
2449 reflections
195 parameters
H atoms treated by a mixture of independent and constrained refinement
Δρ_max_ = 0.52 e Å^−3^
Δρ_min_ = −0.37 e Å^−3^

### 

Data collection: *APEX2* (Bruker, 2009 ▶); cell refinement: *SAINT-Plus* (Bruker, 2009 ▶); data reduction: *SAINT-Plus*; program(s) used to solve structure: *SHELXS97* (Sheldrick, 2008 ▶); program(s) used to refine structure: *SHELXL97* (Sheldrick, 2008 ▶); molecular graphics: *Mercury* (Macrae *et al.*, 2008 ▶); software used to prepare material for publication: *SHELXL97*.

## Supplementary Material

Crystal structure: contains datablock(s) I. DOI: 10.1107/S1600536814001482/hb7189sup1.cif

Structure factors: contains datablock(s) I. DOI: 10.1107/S1600536814001482/hb7189Isup2.hkl

Supporting information file. DOI: 10.1107/S1600536814001482/hb7189Isup3.cml

CCDC reference:

Additional supporting information:  crystallographic information; 3D view; checkCIF report

## Figures and Tables

**Table 1:** Hydrogen-bond geometry (Å, °)

*D*—H⋯*A*	*D*—H	H⋯*A*	*D*⋯*A*	*D*—H⋯*A*
N1—H1⋯O2^i^	0.81 (3)	2.09 (3)	2.872 (3)	172 (3)
C3—H3⋯O1^ii^	0.93	2.57	3.370 (4)	144
C9—H9⋯O2^i^	0.93	2.48	3.288 (3)	145

Symmetry codes: (i) 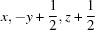; (ii) 
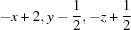
.

**Fig. 1.**
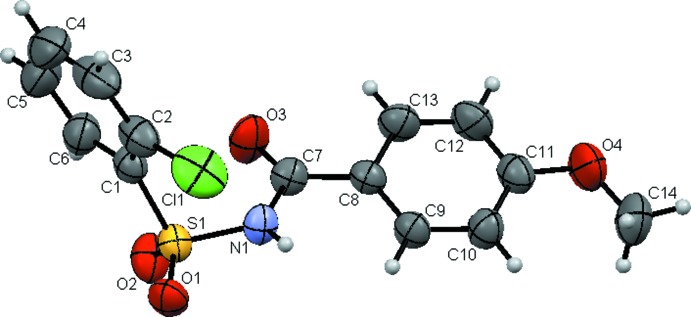
Molecular structure of the title compound, showing displacement ellipsoids drawn at the 50% probability level.

**Fig. 2.**
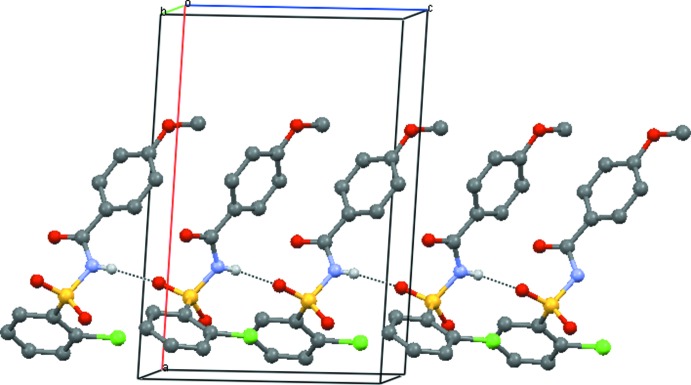
Formation of C(4) chains through N—H···O hydrogen bonds.

**Fig. 3.**
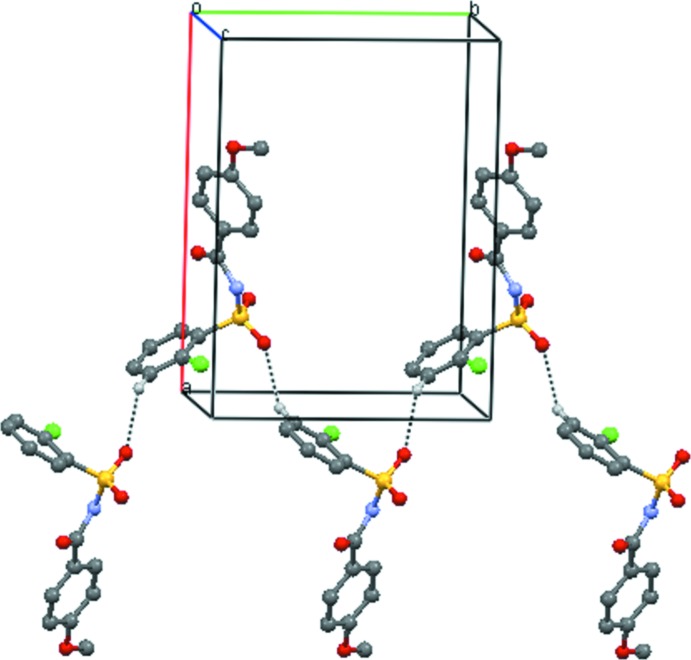
Formation of C(6) chains through C3—H3···O1 interactions running along [001].

**Fig. 4.**
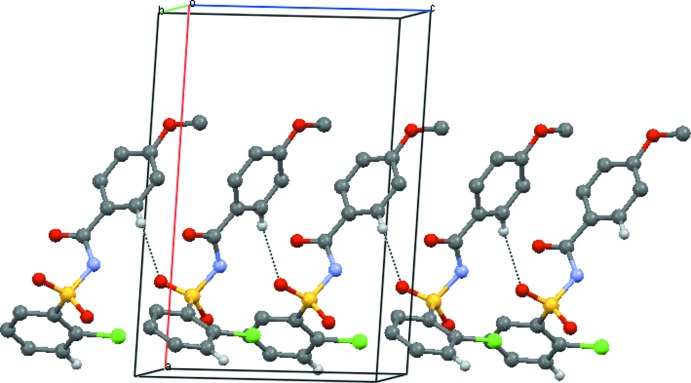
Formation of C(7) chains through C9—H9···O2 interactions running along [001].

**Fig. 5.**
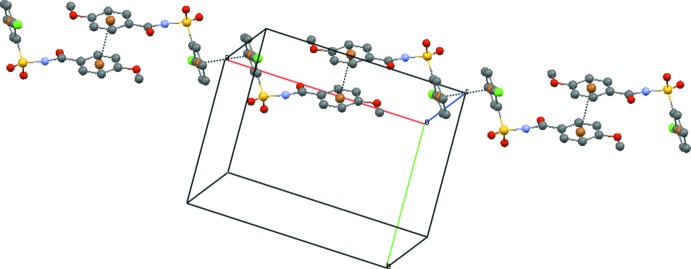
π-π stacking interactions observed in the crystal structure of I.

## supplementary crystallographic information

### 1. Introduction

As a part of our continued structural studies of N-(aroyl)-aryl­sulfonamides (Suchetan *et al.*, 2011*a*, 2011*b*; Gowda *et al.*, 2010*a*, 2010*b*), we report here the crystal structure of the title compound (I) (Fig 1).

### 2.1. Synthesis and crystallization

The title compound (I) was prepared by refluxing a mixture of 4-meth­oxy­benzoic acid, 2-chloro­benzene­sulfonamide and phospho­rous oxychloride (POCl_3_) for 2 h on a water bath. The resultant mixture was cooled and poured into ice cold water. The solid obtained was filtered and washed thoroughly with water and then dissolved in sodium bicarbonate solution. The compound was later reprecipitated by acidifying the filtered solution with dilute HCl. The compound obtained was filtered and later dried (Melting point: 445 K).

Colorless prisms of (I) were obtained from a slow evaporation of its aqueous methano­lic solution at room temperature.

### 2.2. Refinement

The H atom of the NH group was located in a difference map and later refined freely. The other H atoms were positioned with idealized geometry using a riding model with C—H = 0.93-0.96 Å. All H atoms were refined with isotropic displacement parameters (set to 1.2-1.5 times of the U eq of the parent atom).

### 3. Results and discussion

In I, the dihedral angle between the two aromatic rings is 82.07 (1)°. Compared to this, the dihedral angle is 73.3 (1)° in N-(benzoyl)-2-chloro­benzene­sulfonamide (II, Gowda *et al.*, 2010*a*), 85.7 (1)° in N-(4-chloro­benzoyl)-2-chloro­benzene­sulfonamide (III, Suchetan *et al.*, 2011*a*), 89.1 (2)° in N-(4-methyl­benzoyl)-2-chloro­benzene­sulfonamide (IV, Gowda *et al.*, 2010*b*) and 85.4 (1)° in N-(4-nitro­benzoyl)-2-chloro­benzene­sulfonamide (V, Suchetan *et al.*, 2011*b*). This shows that introducing a substituent into the para position of the benzoyl ring of II correlates with a increase of the dihedral angle between the aromatic rings. Further, the molecule is twisted at the S atom, the dihedral angle between the planes defined by the S—N—C=O segment in the central chain and the sulfonyl benzene ring being 82.46 (3)°.

In the crystal structure, the molecules are linked into C(4) chains running along [001] through strong N1—H1···O2 hydrogen bonds (Figure 2). The molecules are further linked into one another through C3—H3···O1 (Figure 3) and C9—H9···O2 (Figure 4) inter­actions into C(6) and C(7) chains running along [001]. The structure is further stabilized by two alternate π-π inter­actions, Cg(meth­oxy­phenyl)-Cg(meth­oxy­phenyl) and Cg(chloro­phenyl)-Cg(chloro­phenyl) distances being respectively 3.577 (1)Å and 3.8016 (1)Å.

### Crystal data

**Table:**

C_14_H_12_ClNO_4_S	Prism
*M**_r_* = 325.76	*D*_x_ = 1.448 Mg m^−^^3^
Monoclinic, *P*2_1_/*c*	Melting point: 445 K
Hall symbol: -P 2ybc	Cu *K*α radiation, λ = 1.54178 Å
*a* = 14.5293 (19) Å	Cell parameters from 25 reflections
*b* = 10.6225 (14) Å	θ = 3.0–64.6°
*c* = 9.6918 (14) Å	µ = 3.71 mm^−^^1^
β = 92.695 (5)°	*T* = 293 K
*V* = 1494.2 (4) Å^3^	Prism, colourless
*Z* = 4	0.44 × 0.35 × 0.26 mm
*F*(000) = 672	

### Data collection

**Table:**

Bruker APEXII CCD diffractometer	2449 independent reflections
Radiation source: fine-focus sealed tube	2295 reflections with *I* > 2σ(*I*)
Graphite monochromator	*R*_int_ = 0.058
phi and φ scans	θ_max_ = 64.6°, θ_min_ = 3.0°
Absorption correction: multi-scan (*SADABS*; Bruker, 2009)	*h* = −16→16
*T*_min_ = 0.263, *T*_max_ = 0.381	*k* = −12→12
17846 measured reflections	*l* = −11→9

### Refinement

**Table:**

Refinement on *F*^2^	Primary atom site location: structure-invariant direct methods
Least-squares matrix: full	Secondary atom site location: difference Fourier map
*R*[*F*^2^ > 2σ(*F*^2^)] = 0.063	Hydrogen site location: inferred from neighbouring sites
*wR*(*F*^2^) = 0.163	H atoms treated by a mixture of independent and constrained refinement
*S* = 1.11	*w* = 1/[σ^2^(*F*_o_^2^) + (0.109*P*)^2^ + 0.3886*P*] where *P* = (*F*_o_^2^ + 2*F*_c_^2^)/3
2449 reflections	(Δ/σ)_max_ < 0.001
195 parameters	Δρ_max_ = 0.52 e Å^−^^3^
0 restraints	Δρ_min_ = −0.37 e Å^−^^3^

### Special details

**Table:**

Geometry. All s.u.'s (except the s.u. in the dihedral angle between two l.s. planes) are estimated using the full covariance matrix. The cell s.u.'s are taken into account individually in the estimation of s.u.'s in distances, angles and torsion angles; correlations between s.u.'s in cell parameters are only used when they are defined by crystal symmetry. An approximate (isotropic) treatment of cell s.u.'s is used for estimating s.u.'s involving l.s. planes.
Refinement. Refinement of *F*^2^ against ALL reflections. The weighted *R*-factor *wR* and goodness of fit *S* are based on *F*^2^, conventional *R*-factors *R* are based on *F*, with *F* set to zero for negative *F*^2^. The threshold expression of *F*^2^ > 2σ(*F*^2^) is used only for calculating *R*-factors(gt) *etc*. and is not relevant to the choice of reflections for refinement. *R*-factors based on *F*^2^ are statistically about twice as large as those based on *F*, and *R*- factors based on ALL data will be even larger.

### Fractional atomic coordinates and isotropic or equivalent isotropic displacement parameters (Å^2^)

**Table:**

	*x*	*y*	*z*	*U*_iso_*/*U*_eq_	
H1	0.718 (2)	0.192 (3)	0.295 (4)	0.066 (10)*	
S1	0.79503 (4)	0.19067 (5)	0.11546 (6)	0.0448 (3)	
Cl1	0.90252 (7)	0.02535 (9)	0.35628 (9)	0.0882 (4)	
O1	0.85786 (12)	0.27002 (19)	0.1915 (2)	0.0592 (5)	
O2	0.75657 (13)	0.2343 (2)	−0.01265 (19)	0.0645 (5)	
O4	0.32115 (14)	0.1078 (2)	0.5017 (3)	0.0826 (7)	
C9	0.54708 (16)	0.1902 (2)	0.3736 (3)	0.0497 (6)	
H9	0.5956	0.2456	0.3921	0.060*	
C8	0.55277 (15)	0.1039 (2)	0.2671 (2)	0.0439 (5)	
C7	0.63122 (16)	0.0964 (2)	0.1759 (2)	0.0467 (6)	
N1	0.71064 (13)	0.1640 (2)	0.2183 (2)	0.0482 (5)	
O3	0.62999 (14)	0.0385 (2)	0.0682 (2)	0.0705 (6)	
C10	0.47035 (18)	0.1952 (3)	0.4531 (3)	0.0562 (7)	
H10	0.4673	0.2543	0.5235	0.067*	
C13	0.47841 (19)	0.0229 (3)	0.2399 (3)	0.0603 (7)	
H13	0.4801	−0.0340	0.1671	0.072*	
C1	0.84769 (15)	0.0440 (2)	0.0838 (3)	0.0485 (6)	
C11	0.39843 (17)	0.1122 (3)	0.4276 (3)	0.0565 (6)	
C2	0.89668 (17)	−0.0230 (3)	0.1868 (3)	0.0604 (7)	
C12	0.4026 (2)	0.0265 (3)	0.3197 (4)	0.0679 (8)	
H12	0.3539	−0.0287	0.3013	0.081*	
C3	0.9432 (2)	−0.1334 (3)	0.1497 (5)	0.0842 (12)	
H3	0.9776	−0.1787	0.2160	0.101*	
C6	0.8439 (2)	0.0008 (3)	−0.0515 (3)	0.0638 (7)	
H6	0.8105	0.0453	−0.1195	0.077*	
C4	0.9373 (3)	−0.1740 (3)	0.0131 (5)	0.0893 (12)	
H4	0.9668	−0.2481	−0.0107	0.107*	
C5	0.8897 (3)	−0.1082 (4)	−0.0854 (5)	0.0851 (11)	
H5	0.8877	−0.1362	−0.1765	0.102*	
C14	0.3152 (3)	0.1932 (4)	0.6139 (5)	0.0940 (13)	
H14A	0.3676	0.1823	0.6768	0.141*	
H14B	0.2598	0.1771	0.6612	0.141*	
H14C	0.3141	0.2780	0.5795	0.141*	

### Atomic displacement parameters (Å^2^)

**Table:**

	*U*^11^	*U*^22^	*U*^33^	*U*^12^	*U*^13^	*U*^23^
S1	0.0411 (4)	0.0523 (4)	0.0410 (4)	0.0020 (2)	0.0010 (3)	0.0046 (2)
Cl1	0.0929 (6)	0.0998 (7)	0.0690 (6)	−0.0016 (5)	−0.0274 (4)	0.0253 (4)
O1	0.0552 (10)	0.0589 (10)	0.0636 (12)	−0.0150 (8)	0.0034 (8)	−0.0062 (8)
O2	0.0623 (11)	0.0852 (13)	0.0459 (10)	0.0168 (10)	0.0013 (8)	0.0179 (9)
O4	0.0488 (11)	0.0906 (16)	0.1105 (19)	−0.0120 (10)	0.0257 (11)	−0.0087 (13)
C9	0.0382 (12)	0.0555 (14)	0.0552 (15)	−0.0069 (9)	−0.0011 (10)	−0.0066 (10)
C8	0.0404 (11)	0.0450 (12)	0.0459 (12)	−0.0027 (9)	−0.0037 (9)	0.0013 (10)
C7	0.0436 (12)	0.0541 (13)	0.0418 (13)	0.0007 (10)	−0.0048 (9)	−0.0023 (10)
N1	0.0421 (10)	0.0628 (12)	0.0394 (12)	−0.0026 (9)	−0.0010 (8)	−0.0064 (10)
O3	0.0594 (11)	0.0961 (15)	0.0557 (12)	−0.0101 (10)	0.0010 (9)	−0.0290 (11)
C10	0.0429 (13)	0.0642 (16)	0.0616 (16)	−0.0030 (10)	0.0047 (11)	−0.0110 (12)
C13	0.0529 (14)	0.0595 (15)	0.0681 (17)	−0.0110 (12)	−0.0015 (12)	−0.0141 (13)
C1	0.0368 (11)	0.0534 (13)	0.0555 (14)	−0.0019 (9)	0.0043 (10)	0.0023 (11)
C11	0.0406 (12)	0.0594 (15)	0.0697 (17)	−0.0029 (11)	0.0055 (11)	0.0053 (13)
C2	0.0396 (12)	0.0604 (16)	0.0806 (19)	−0.0003 (11)	−0.0018 (12)	0.0167 (13)
C12	0.0467 (14)	0.0664 (17)	0.091 (2)	−0.0180 (12)	0.0039 (13)	−0.0085 (15)
C3	0.0488 (15)	0.0647 (19)	0.139 (4)	0.0075 (13)	0.0061 (18)	0.030 (2)
C6	0.0598 (15)	0.0699 (17)	0.0627 (17)	−0.0006 (13)	0.0115 (13)	−0.0087 (14)
C4	0.072 (2)	0.066 (2)	0.133 (4)	0.0070 (16)	0.032 (2)	−0.012 (2)
C5	0.081 (2)	0.077 (2)	0.100 (3)	−0.0035 (18)	0.030 (2)	−0.025 (2)
C14	0.0640 (19)	0.103 (3)	0.119 (3)	0.0030 (18)	0.041 (2)	−0.016 (2)

### Geometric parameters (Å, º)

**Table:**

S1—O2	1.4154 (19)	C13—C12	1.375 (4)
S1—O1	1.4227 (19)	C13—H13	0.9300
S1—N1	1.641 (2)	C1—C6	1.387 (4)
S1—C1	1.769 (3)	C1—C2	1.394 (4)
Cl1—C2	1.720 (3)	C11—C12	1.390 (5)
O4—C11	1.362 (3)	C2—C3	1.408 (5)
O4—C14	1.422 (5)	C12—H12	0.9300
C9—C10	1.385 (4)	C3—C4	1.391 (6)
C9—C8	1.386 (4)	C3—H3	0.9300
C9—H9	0.9300	C6—C5	1.383 (5)
C8—C13	1.396 (3)	C6—H6	0.9300
C8—C7	1.477 (3)	C4—C5	1.348 (6)
C7—O3	1.211 (3)	C4—H4	0.9300
C7—N1	1.404 (3)	C5—H5	0.9300
N1—H1	0.80 (4)	C14—H14A	0.9600
C10—C11	1.381 (4)	C14—H14B	0.9600
C10—H10	0.9300	C14—H14C	0.9600
			
O2—S1—O1	118.55 (13)	O4—C11—C10	124.3 (3)
O2—S1—N1	108.28 (11)	O4—C11—C12	116.2 (2)
O1—S1—N1	105.51 (11)	C10—C11—C12	119.6 (2)
O2—S1—C1	106.98 (13)	C1—C2—C3	118.5 (3)
O1—S1—C1	109.85 (12)	C1—C2—Cl1	122.2 (2)
N1—S1—C1	107.16 (11)	C3—C2—Cl1	119.3 (3)
C11—O4—C14	117.6 (2)	C13—C12—C11	120.4 (2)
C10—C9—C8	121.2 (2)	C13—C12—H12	119.8
C10—C9—H9	119.4	C11—C12—H12	119.8
C8—C9—H9	119.4	C4—C3—C2	119.5 (3)
C9—C8—C13	118.4 (2)	C4—C3—H3	120.2
C9—C8—C7	123.8 (2)	C2—C3—H3	120.2
C13—C8—C7	117.8 (2)	C5—C6—C1	120.3 (3)
O3—C7—N1	119.4 (2)	C5—C6—H6	119.9
O3—C7—C8	124.2 (2)	C1—C6—H6	119.9
N1—C7—C8	116.4 (2)	C5—C4—C3	121.3 (3)
C7—N1—S1	122.55 (18)	C5—C4—H4	119.4
C7—N1—H1	122 (2)	C3—C4—H4	119.4
S1—N1—H1	115 (2)	C4—C5—C6	120.1 (4)
C11—C10—C9	119.9 (2)	C4—C5—H5	119.9
C11—C10—H10	120.1	C6—C5—H5	119.9
C9—C10—H10	120.1	O4—C14—H14A	109.5
C12—C13—C8	120.6 (3)	O4—C14—H14B	109.5
C12—C13—H13	119.7	H14A—C14—H14B	109.5
C8—C13—H13	119.7	O4—C14—H14C	109.5
C6—C1—C2	120.3 (3)	H14A—C14—H14C	109.5
C6—C1—S1	117.2 (2)	H14B—C14—H14C	109.5
C2—C1—S1	122.4 (2)		
			
C10—C9—C8—C13	1.0 (4)	N1—S1—C1—C2	−67.4 (2)
C10—C9—C8—C7	178.0 (2)	C14—O4—C11—C10	−1.0 (5)
C9—C8—C7—O3	−165.9 (3)	C14—O4—C11—C12	179.3 (3)
C13—C8—C7—O3	11.1 (4)	C9—C10—C11—O4	178.5 (3)
C9—C8—C7—N1	12.8 (3)	C9—C10—C11—C12	−1.8 (4)
C13—C8—C7—N1	−170.2 (2)	C6—C1—C2—C3	1.1 (4)
O3—C7—N1—S1	12.0 (3)	S1—C1—C2—C3	−174.5 (2)
C8—C7—N1—S1	−166.74 (17)	C6—C1—C2—Cl1	−179.3 (2)
O2—S1—N1—C7	47.2 (2)	S1—C1—C2—Cl1	5.1 (3)
O1—S1—N1—C7	175.11 (19)	C8—C13—C12—C11	0.9 (5)
C1—S1—N1—C7	−67.9 (2)	O4—C11—C12—C13	−179.3 (3)
C8—C9—C10—C11	0.8 (4)	C10—C11—C12—C13	0.9 (5)
C9—C8—C13—C12	−1.9 (4)	C1—C2—C3—C4	−1.5 (4)
C7—C8—C13—C12	−179.0 (3)	Cl1—C2—C3—C4	178.9 (3)
O2—S1—C1—C6	0.9 (2)	C2—C1—C6—C5	−0.8 (4)
O1—S1—C1—C6	−129.0 (2)	S1—C1—C6—C5	175.0 (2)
N1—S1—C1—C6	116.9 (2)	C2—C3—C4—C5	1.6 (5)
O2—S1—C1—C2	176.7 (2)	C3—C4—C5—C6	−1.3 (6)
O1—S1—C1—C2	46.8 (2)	C1—C6—C5—C4	0.9 (5)

### Hydrogen-bond geometry (Å, º)

**Table:**

*D*—H···*A*	*D*—H	H···*A*	*D*···*A*	*D*—H···*A*
N1—H1···O2^i^	0.81 (3)	2.09 (3)	2.872 (3)	172 (3)
C3—H3···O1^ii^	0.93	2.57	3.370 (4)	144
C9—H9···O2^i^	0.93	2.48	3.288 (3)	145

Symmetry codes: (i) *x*, −*y*+1/2, *z*+1/2; (ii) −*x*+2, *y*−1/2, −*z*+1/2.
